# S100A8/A9 increases the mobilization of pro-inflammatory Ly6C^high^ monocytes to the synovium during experimental osteoarthritis

**DOI:** 10.1186/s13075-017-1426-6

**Published:** 2017-09-29

**Authors:** Niels A. J. Cremers, Martijn H. J. van den Bosch, Stephanie van Dalen, Irene Di Ceglie, Giuliana Ascone, Fons van de Loo, Marije Koenders, Peter van der Kraan, Annet Sloetjes, Thomas Vogl, Johannes Roth, Edwin J. W. Geven, Arjen B. Blom, Peter L. E. M. van Lent

**Affiliations:** 1 Experimental Rheumatology, Department of Rheumatology, Radboud University Medical Center, PO Box 9101, Nijmegen, 6500 HB The Netherlands; 2 Institute of Immunology, University of Munster, Munster, Germany

**Keywords:** S100, Mobilization, Ly6C high/low monocytes, Systemic effects, Collagenase-induced osteoarthritis

## Abstract

**Background:**

Monocytes are dominant cells present within the inflamed synovium during osteoarthritis (OA). In mice, two functionally distinct monocyte subsets are described: pro-inflammatory Ly6C^high^ and patrolling Ly6C^low^ monocytes. Alarmins S100A8/A9 locally released by the synovium during inflammatory OA for prolonged periods may be dominant proteins involved in stimulating recruitment of Ly6C^high^ monocytes from the circulation to the joint. Our objective was to investigate the role of S100A8/A9 in the mobilization of Ly6C^high^ and Ly6C^low^ monocytic populations to the inflamed joint in collagenase-induced OA (CiOA).

**Method:**

S100A8 was injected intra-articularly to investigate monocyte influx. CiOA was induced by injection of collagenase into knee joints of wild-type C57BL/6 (WT), and S100a9^-/-^ mice. Mice were sacrificed together with age-matched saline-injected control mice (n = 6/group), and expression of monocyte markers, pro-inflammatory cytokines, and chemokines was determined in the synovium using ELISA and RT-qPCR. Cells were isolated from the bone marrow (BM), spleen, blood, and synovium and monocytes were identified using FACS.

**Results:**

S100A8/A9 was highly expressed during CiOA. Intra-articular injection of S100A8 leads to elevated expression of monocyte markers and the monocyte-attracting chemokines CCL2 and CX3CL1 in the synovium. At day 7 (d7) after CiOA induction in WT mice, numbers of Ly6C^high^, but not Ly6C^low^ monocytes, were strongly increased (7.6-fold) in the synovium compared to saline-injected controls. This coincided with strong upregulation of CCL2, which preferentially attracts Ly6C^high^ monocytes. In contrast, S100a9^-/-^ mice showed a significant increase in Ly6C^low^ monocytes (twofold) within the synovium at CiOA d7, whereas the number of Ly6C^high^ monocytes remained unaffected. In agreement with this finding, the Ly6C^low^ mobilization marker CX3CL1 was significantly higher within the synovium of S100a9^-/-^ mice. Next, we studied the effect of S100A8/A9 on release of Ly6C^high^ monocytes from the BM into the circulation. A 14% decrease in myeloid cells was found in WT BM at CiOA d7. No decrease in myeloid cells in S100a9^-/-^ BM was found, suggesting that S100A8/A9 promotes the release of myeloid populations from the BM.

**Conclusion:**

Induction of OA locally leads to strongly elevated S100A8/A9 expression and an elevated influx of Ly6C^high^ monocytes from the BM to the synovium.

**Electronic supplementary material:**

The online version of this article (doi:10.1186/s13075-017-1426-6) contains supplementary material, which is available to authorized users.

## Background

Osteoarthritis (OA) is a chronic degenerative disease of the joints and currently its high prevalence increases even further due to aging and an increasingly obese population [1–3]. Current treatment options focus on targeting the symptoms and are limited to analgesics and anti-inflammatory drugs, with many patients eventually having to undergo joint replacement surgery [4–6]. Novel therapies that focus on preventing joint damage are warranted. A better insight into the etiology and disease progression is therefore needed [3, 4].

The etiology of OA is multi-factorial, and it is considered to be a disease of the whole joint in which the synovium also plays an important role [7–9]. Synovial activation is clearly present in more than 50% of patients with OA and contributes to the pathophysiology and clinical symptoms [6–10]. Systemic and local synovial inflammation is increasingly recognized to be involved in joint pathologic change [6, 11, 12]. Although most types of leucocytes have been described to be present within the inflamed OA synovium, monocytes and macrophages are thought to be the predominant cell types driving the pathologic change [13–15]. OA is characterized by joint damage, which leads to the release of proteins or alarmins such as S100A8/A9 promoting activation of monocytes/macrophages within the synovium followed by an inherent release of inflammatory cytokines, such as IL-1β, TNF-α and additional release of S100 alarmins [7, 9, 10, 16]. The most prominent proteins from the S100 family that are released during OA are S100A8 (myeloid-related proteins: MRP8) and S100A9 (MRP14), which belong to the group of damage-associated molecular patterns proteins (DAMPs) or alarmins, and play a crucial role in innate immunity [17–19]. S100A8 and S100A9 form heterodimers under low calcium conditions within the cell and assemble into (S100A8/A9)^2^ hetero-tetramers in the presence of calcium [20]. When myeloid cells are stressed S100A8/A9 is secreted and binds to the toll-like receptor (TLR)4 receptor promoting pro-inflammatory effects [19, 21]. We previously described that S100A8/A9 proteins are expressed for prolonged periods in collagenase-induced OA (CiOA), a model driven by synovial inflammation, and that they are important stimulators of tissue pathology [22–24]. In addition, patients with OA are characterized by high levels of S100A8/A9 in blood and synovium, and baseline serum levels of patients with symptomatic OA predict development of joint destruction 2 years thereafter [22, 23]. Moreover, S100A8/A9 promotes the migration of monocytes, which when activated, are also important producers of S100A8/A9 thereby forming a positive feedback loop [25, 26].

Monocytes are recruited from the bone marrow (BM) towards the site of inflammation via a combined action of adhesion molecules, e.g. LFA-1 and VCAM, and chemokines, such as (C-C motif) ligand 2 (CCL2) that is known to bind the C-C chemokine receptor type 2 (CCR) [27–30]. In mice, two functionally distinct monocyte subpopulations are described. The pro-inflammatory Ly6C^high^ monocytes, which express high levels of CCR2, are involved in removing debris, and the Ly6C^low^ monocytes, which express high levels of CX3CR1, are suggested to be involved in repair processes as they release anabolic factors like vascular endothelial growth factor (VEGF) and transforming growth factor (TGF)-β [31, 32]. Ly6C^high^ monocytes reside in the BM and upon release from the BM into the circulation can either stay in their state or transform into Ly6C^low^ monocytes [32, 33]. Once in the blood, both Ly6C^high^ as Ly6C^low^ monocytes can be mobilized to the peripheral tissue in response to chemokines [34]. Ly6C^high^ monocytes are mainly attracted by CCL2 and Ly6C^low^ monocytes by CX3CL1, which can bind to CCR2 and CX3CR1, respectively [35]. When the monocytes arrive at the injured site, they can differentiate into M1-like pro-inflammatory or M2-like anti-inflammatory macrophages, dependently on the environmental cues [33]. The aim of our study is to investigate whether local production of S100A8/A9 in the inflamed joint and their subsequent release into the circulation is involved in recruitment of the different monocyte cell populations to the joint in inflammatory CiOA.

## Methods

### Animals

The Committee for Animal Experiments of the Dutch Central Commission on Animal Experiments approved all procedures involving animals (CCD# 2015-0014). One hundred fifty-eight female mice (strain: 106 C57BL/6 and 52 S100a9^-/-^), 8–12 weeks of age, and weighing 21 ± 1.5 g (minimum 19 g, maximum 26 g) were provided with standard diet and water *ad libitum* and maintained on a 12-h light/dark cycle under specific pathogen-free housing conditions at the Central Animal Facility Nijmegen. More details on the housing conditions have been previously described [36]. S100a9^-/-^ mice were originally generated at the University of Münster as previously described [37]. An overview of the animals used for the different experiments and analyzed time-points can be found in Table 1. No animals died during the experiments and no animals were excluded during the experiments or data analysis. All mice were randomly divided over the experiments and different conditions. All outcomes were measured by an observer who was blinded to the allocation of the animals to the experimental groups, when possible. The mice were anesthetized with 5% isoflurane in O_2_/N_2_O and killed by exsanguination, followed by cervical dislocation. At the end of the experiment, at day 7, 21, or 42 after induction of CiOA, different tissues (synovium, blood, and contralateral femur) were collected and used for histologic evaluation, collection of serum for protein analysis, measurement of messenger RNA (mRNA) expression, and determination of different cell populations.

**Table 1:** Overview of animals used in experiments

Experiment	Readout	Time point	Number of animals per group and strains
CiOA/control	Monocyte populations and RNA expression synovium and BM	Day 7	6 CiOA/6 control C57BL/6
			6 CiOA/6 control S100a9^-/-^
CiOA/control	BM count	Day 7, 21, and 42	6 CiOA/7 control C57BL/6 (day 7)
			6 CiOA/7 control C57BL/6 (day 21)
			7 CiOA/7 control C57BL/6 (day 42)
CiOA/control	Monocyte populations and BM count	Day 7	5 CiOA/5 control C57BL/6
			5 CiOA/5 control S100a9^-/-^
CiOA	Immunohistologic evaluation (and serum for protein analysis)	Day 7, 21, and 42	10 per time-point C57BL/6 (day 7)
			10 per time-point S100a9^-/-^ (day 7)
			10 per time-point C57BL/6 (day 21)
			10 per time-point S100a9^-/-^ (day 21)
			10 per time-point C57BL/6 (day 42)
			10 per time-point S100a9^-/-^ (day 42)
i.a. injection with S100A8/BSA	Immunohistologic evaluation	Day 1	8 C57BL/6
i.a. injection with S100A8/BSA	RNA expression	Day 1	6 C57BL/6

*CiOA* collagenase-induced osteoarthritis, *i.a*., intra-articular, *B*M bone marrow

### Collagenase induced OA model and tissue isolation

CiOA was unilaterally induced in wild-type (WT) C57BL/6 mice by injection of one unit of collagenase type VII from *Clostridium histolyticum* (Sigma Chemical Co., St. Louis, MO, USA) in a volume of 6 μL twice on alternate days in the right knee joint, as previously described [38]. An equal volume of saline was injected into the right knee of age-matched mice and served as the treated control. Mice were killed at different time points (day 7, 21, or 42) after CiOA induction, and the different readouts as described in Table 1 were determined. In short, mRNA expression (reverse transcription quantitative PCR (RT-qPCR)) and protein levels (Luminex/ELISA) of mobilization markers and pro-inflammatory cytokines, including S100A8 and S100A9, were measured in the synovium and BM cells isolated from the contralateral femur. Synovial biopsies were taken using a 3-mm-diameter biopsy punch. The absolute number of cells in the contralateral BM per femur was determined by crushing the complete femur with a mortar and pestle, intensive lavation with PBS, and counting of the cells. Monocyte subpopulations of BM cells present in the contralateral femur, and CiOA-affected synovial tissue were analyzed by FACS. Tissue sections were stained with hematoxylin and eosin (HE) and histologically scored for synovial activation (number of cell layers of synovial intima lining), and immunohistochemically stained for S100A8, S100A9 and Ly6C.

### Intra-articular injection of S100A8 protein

Mice were injected intra-articularly with a volume of 6 μL PBS containing 5 μg of S100A8 recombinant protein in the right knee and 5 μg bovine serum albumin (BSA) protein as a control in the left knee, after which the mice were killed and synovium was isolated for histologic evaluation and for RT-qPCR analysis as described in Table 1.

### RNA isolation and RT-qPCR

Total RNA from BM cells was isolated using TRIzol reagent according to the manufacturer’s protocol (Invitrogen). Cells of the synovial tissue were lyzed and homogenized using ceramic MagNa Lyser Green Beads (Roche) and the MagNa Lyser (Roche) three times for 20 s at 6000 rpm with 1 min interspersed cooling. RNA of the synovium was further extracted with the RNeasy Fibrous Tissue mini kit (Qiagen, catalog (cat) # 74704, Venlo, The Netherlands). DNase treatment (Qiagen: RNase-Free DNase Set) was performed between the first washing steps with RW1 buffer. RNA concentration was determined using the nanodrop 2000 spectrophotometer (Thermo Scientific). For the reverse transcriptase treatment, 1 μg sample RNA was linearized in a volume of 11 μL water for 15 min at 65 °C and then together with total reverse transcriptase mix (containing 100 μM DTT, 10 mM dNTP each, 0.2 μg oligodT primer, 20 units RNA inhibitor (RNAsin: Promega cat # N251A), and 200 units reverse transcriptase (Invitrogen 28025-013)) in a total volume of 20 μL incubated for 5 min at 25 °C, 60 min at 39 °C and 5 min at 94 °C. Hereafter the complementary DNA (cDNA) was diluted × 20 and used for RT-qPCR, using the StepOnePlus Real-Time PCR System (Applied Biosystems). The reaction was performed in a volume of 10 μL containing 3 μL cDNA, 0.6 μM primers, 5 μL iQ SYBR Green Supermix (Bio-Rad Laboratories). After incubation of 3 min, amplification was carried out for 40 cycles of 15 s at 95 °C and 30 s at 60 °C. The melting temperature of the products was defined to indicate amplification specificity. All values were normalized to the reference gene *Gapdh*, which is often used in CiOA experiments and showed stable expression during this model, and was presented as –Δ cycle threshold (–ΔCt) [39]. We decided to present the negative ΔCt (Ct of the reference gene minus the Ct of the gene of interest) to improve the ease of interpretation (higher values in the graphs representing higher expression). Fold change can be calculated as the difference in − ΔCt (giving ΔΔCt) between saline vs CiOA or WT vs S100a9^-/-^ groups, and then 2^ΔΔCt^. Primers are summarized in Table 2. All primers were custom-designed using Primer-BLAST (https://www.ncbi.nlm.nih.gov/tools/primer-blast/) at standard settings with a product size of 70–150 bp, spanning an exon-exon junction, max poly-X at 4, max GC in primer 3’ end at 3, and primer GC content between 20 and 80%, and subsequently validated in serial dilutions of mice cDNA to test the amplification efficiency of the RT-qPCR. For an efficiency of 100%, the slope is − 3.32; all our primers passed the minimal efficiency requirements of 90–110%, corresponding with a slope between − 3.58 and − 3.1. All primers were checked for specificity by performing a melt curve, confirming a single product after the PCR assay and no formation of aspecific by-products.

**Table 2:** Primer sequences of mouse inflammatory and mobilization markers

	Sense	Antisense
Reference gene		
*Gapdh*	5′-ggcaaattcaacggcaca-3′	5′-gttagtggggtctcgctcctg-3′
Inflammation marker		
*S100A8*	5′-tgtcctcagtttgtgcagaatataaat-3′	5′-tttatcaccatcgcaaggaactc-3′
*S100A9*	5′-ggcaaaggctgtgggaagt-3′	5′-ccattgagtaagccattcccttta-3′
Mobilization markers		
*CCL2*	5′-ttggctcagccagatgca-3′	5′-cctactcattgggatcatcttgct-3′
*CX3CL1*	5′-gtgccattgtcctggagac-3′	5′-catttctccttcgggtcag-3′
*KC*	5′-tggctgggattcacctcaa-3′	5′-gagtgtggctatgacttcggttt-3′
*MIP*-*1α*	5′-caagtcttctcagcgccatatg-3′	5′-tcttccggctgtaggagaagc-3′
Cell markers		
*CX3CR1*	5′-gagtatgacgattctgctgagg-3′	5′-cagaccgaacgtgaagacgag-3′
*CCR2*	5′-ctatctgctcaacttggccatct-3′	5′-tgagcccagaatggtaatgtga-3′
*Ly6C*	5′-gcagtgctacgagtgctatgg-3′	5′-actgacgggtctttagtttcctt-3′
*F4*/*80*	5′-aatcctgtgaagatgtgg-3′	5′-gagtgttgatgcaaatgaag-3′
Adhesion molecules		
*LFA1*	5′-gaatgtatgaagggcaaagtc-3′	5′-gcagcaaactggtaggaag-3′
*VCAM*	5′-ccaagtctctccaaaagatatacagctt-3′	5′-atgacggtgtctccctct-3′
*L*-*selectin*	5′-actgctctgttgtgacttcc-3′	5′-tgtatggcgactaaatctgtg-3′
*PECAM1*	5′-tccctgggaggtcgtccat-3′	5′-gaacaaggcagcggggttta-3′
*VE*-*cadherin*	5′-tcctcttgcatcctcactatcaca-3′	5′-gtaagtgaccaactgctcgtgaat-3′

### Histologic evaluation of total knee joints

Dissected total knee joints were fixed in 4% formalin, decalcified in 5% formic acid, and subsequently embedded in paraffin. Coronal knee joint sections of 7 μm thickness were made and mounted on superfrost glass slides (SuperFrost® Plus, Menzel-Glaser, Germany). Deparaffinized sections were stained with HE. Synovial activation was evaluated by two blinded observers scoring the thickening of the synovial lining layer (number of cell layers of the intima) and the cellular influx as described previously [40].

### Immunohistochemical staining of S100A8, S100A9, and Ly6C

Knee joints sections were stained for S100A8, S100A9, and Ly6C as described previously [22]. In short, antigen retrieval was performed by incubating for 15 min in citrate buffer at 60 °C. Thereafter, sections were incubated for 1 h with the primary antibodies directed against mouse S100A8 (host, rabbit), mouse S100A9 (host, rabbit) (both were made at our own facilities [41], and used at a concentration of 1 μg/mL), or mouse Ly6C (host, rat) (Abcam cat # 15627, at a concentration at 2 μg/mL). Rabbit (for S100A8 and S100A9) or rat (for Ly6C) IgG antibody was used as a control. After washing, S100A8 and S100A9 sections were incubated with horseradish peroxidase (HRP)-conjugated goat anti-rabbit IgG (Dako, Glostrup, Denmark) for 30 min. For the Ly6C staining, after incubation with the primary antibody, and washing, sections were incubated with secondary rabbit anti rat IgG biotin-labeled antibody for 30 min, followed by washing and a 30-min incubation with VECTASTAIN elite ABC HRP kit (Peroxidase, Rabbit IgG) (Vector laboratories, Burlingame, CA, USA, cat # PK6101). Sections were peroxidase-stained using diaminobenxidine and counterstained using Mayer’s hemotoxylin (Biochemica, Amsterdam, the Netherlands).

### Determination of monocyte subpopulations using flow cytometry

Cells isolated from the BM and synovial tissues were analyzed for monocyte subpopulations by seven-color staining and flow cytometry using the Gallios flow cytometer (Beckman Coulter, Indianapolis, IN, USA). The antibodies and fluorophores that were used are summarized in Table 3. First, single cells were selected using the side scatter and pulse width. Next, viable cells were gated using the side scatter and negative for the SYTOX blue viability staining. Myeloid cells, were gated negative for the dump channel (CD90/B220/CD49b/NK1.1) to deplete T cells, B cells, and natural killer (NK) cells, and positively selected for CD11b. Cells were next plotted for Ly6G and Ly6C to distinguish neutrophils and monocytes, respectively. Monocytes were selected and finally plotted for F4/80 and Ly6C. Herein, Ly6C^high^ and Ly6C^low^ monocytes and macrophages could be selected dependent on the tissue. Monocyte subsets were identified as (B220/CD90/CD49b/NK1.1/Ly6G)^low^CD11b^high^(F4/80/MHCII/CD11c)^low^ and further distinguished by their Ly6C expression, using Kaluza flow cytometry analysis software (Beckman Coulter).

**Table 3:** Antibodies used to detect monocyte subsets using flow cytometry

Marker	Fluorophore	Manufacturer (catalog number)
CD11b	FITC	BD Biosciences (553310)
Ly6C	APC-Cy7	BD Biosciences (560596)
Ly6G	APC	Biolegend (127614)
CD90.2	PE	BD Biosciences (553006)
B220/antiCD45	PE	BD Biosciences (553090)
CD49b	PE	BD Biosciences (553858)
NK1.1	PE	BD Biosciences (553165)
F4/80	PE-Cy7	Biolegend (123114)
MHCII	PE-Cy7	Biolegend (116420)
CD11c	PE-Cy7	BD Biosciences (558079)
SYTOX Blue Dead Cell Stain	SYTOX blue	Thermo Fisher Scientific (S34857)

### Statistics

Data were analyzed using GraphPad Prism 5.01 software (San Diego, CA, USA). Outliers were tested using Grubbs’ test, but no outliers were found. Data were analyzed using the two-sided *t* test to compare two variables or one-way analysis of variance when comparing multiple variables. Bonferroni’s multiple comparison post hoc test was applied as correction for multiple comparisons when investigating multiple dependent research questions. Results were considered significantly different at *p* < 0.05 (with significance level denoted as *p < 0.05, **p < 0.01, and ***p < 0.001).

## Results

### Increased numbers of monocytes in the synovium after induction of CiOA coincides with high levels of S100A8/A9

**Fig. 1:**
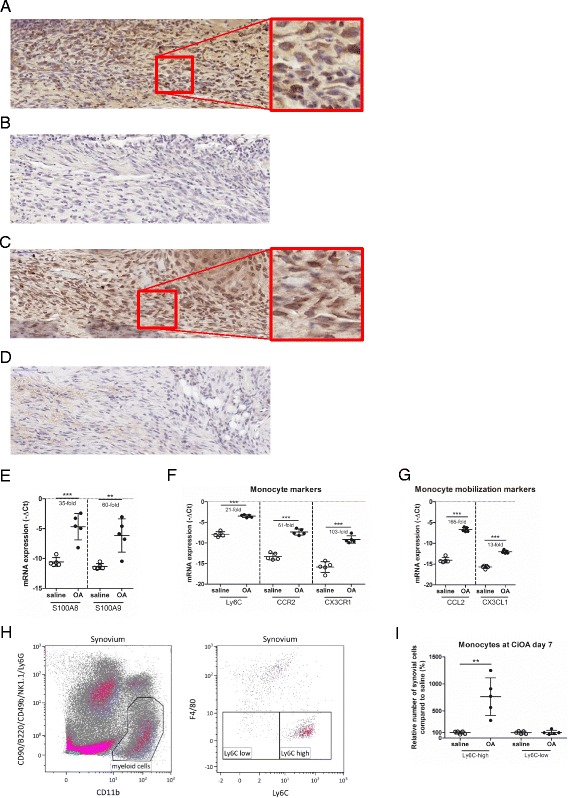
S100A8/A9 mRNA and protein expression is strongly increased in the synovium at day 7 of collagenase-induced osteoarthritis (CiOA), leading to elevated Ly6C^high^ monocytes locally into the joint. **a** Monocytes are strongly infiltrated during early CiOA as shown by highly expressed Ly6C protein staining and based on cell morphology in the synovial lining layer of the knee joints at CiOA day 7. **b** IgG control staining for the Ly6C antibody of the same joint shows no aspecific staining. **c** S100A9 protein is expressed in synovial monocytes at CiOA day 7 as shown by immuno-localization. **d** IgG control staining for the S100A9 antibody of the same joint shows no aspecific staining. **e** S100A8 and S100A9 mRNA expression is strongly induced in the synovium at CiOA day 7 when compared to saline-injected controls. **f**, **g** Monocyte marker Ly6C, and the more monocyte-subset-specific markers, CCR2 (Ly6C^high^) and CX3CR1 (Ly6C^low^) are strongly induced (**f**), together with the monocyte attracting chemokines CCL2 (Ly6C^high^) and CX3CL1 (Ly6C^low^) (**g**) in synovium at CiOA day 7, when compared to saline-injected controls. **h** Monocyte gating strategy for flow cytometric analysis: single viable cells were plotted for B220/CD90/CD49b/NK1.1/Ly6G and CD11b, in which myeloid cells were selected, and next the monocyte subsets could be identified based on their F4/80/MHCII/CD11c and Ly6C expression. **i** The relative number of Ly6C^high^ monocytes were increased, whereas Ly6C^low^ monocyte subpopulations remained unaffected locally in the joint at CiOA day 7 compared to saline-injected controls, as observed using flow cytometry. Data represent mean ± SD of five individual mice. *Significantly different from saline-injected control (**p < 0.01, ***p < 0.001). Ct cycle threshold

We first investigated whether monocytes are attracted to the inflamed joint in experimental inflammatory OA. At day 7 after induction of CiOA, the number of cell layers in the intima-lining layer was increased and mainly consisted of leucocytes with monocyte morphology. Most of the cells within the synovial lining strongly expressed Ly6C, a protein characteristic of monocytes, as shown by immuno-localization (Fig. 1a vs control b).

Next we studied whether S100A8/A9 expression was related to this increase in monocyte numbers within the inflamed joint. mRNA levels of S100A8 and S100A9 were strongly enhanced (35-fold and 60-fold, respectively) at CiOA day 7, when compared to saline-injected controls (Fig. 1e). Immuno-localization shows that S100A8/A9 protein was strongly expressed by monocytes within the inflamed synovial lining (Fig. 1c vs control d). At later time points after CiOA, S100A8/A9 levels were still high whereas in contrast IL-1β, TNF-α and IL-6 rapidly decreased [22], suggesting that synovial inflammation in CiOA is dominated by S100A8/A9 production.

To analyze the presence of pro-inflammatory Ly6C^high^ monocytes and reparative Ly6C^low^ monocyte subsets, we next investigated mRNA expression within the inflamed synovium of chemokine receptors associated with specific monocyte subsets (Fig. 1f). Ly6C^high^ monocytes are characterized by expression of mainly CCR2 and to a lesser extent CX3CR1, whereas Ly6C^low^ monocytes predominantly express CX3CR1. mRNA expression levels of Ly6C, CCR2, and CX3CR1 were all strongly increased within the inflamed synovium of early-phase CiOA (day 7) (21-fold, 61-fold, and 103-fold, respectively) (Fig. 1f). Next we analyzed monocyte-subset-attracting chemokines (Fig. 1g). CCL2 (MCP-1) is dominant for Ly6C^high^ monocytes, whereas CX3CL1 particularly attracts Ly6C^low^ monocytes. mRNA expression levels of CCL2 were strongly increased (165-fold), whereas CX3CL1 was only increased 13-fold (Fig. 1g). The strongly increased expression of both CCR2 and its ligand CCL2 within the inflamed synovium suggests an elevated presence of Ly6C^high^ monocytes.

### Specifically, Ly6C^high^ monocytes are increased in the synovium after induction of CiOA

To analyze more accurately which monocyte subsets are indeed preferentially increased within the inflamed synovium during the first phase of CiOA, we treated synovial tissue obtained from mice with CiOA and saline-injected control mice with collagenase and analyzed the cell composition by flow cytometry. Single viable cells were selected, after which myeloid cells were identified as CD11b^high^ and B220/CD90/CD49b/NK1.1/Ly6G^low^ to exclude B cells, T cells, NK -cells, and neutrophils. Next, monocytes subsets can be further divided based on their Ly6C expression (Fig. 1h). In synovium at CiOA day 7, the number of Ly6C^high^ monocytes was significantly increased (764% increase) in contrast to Ly6C^low^ monocytes, which were not different in synovium obtained from saline-injected control mice (Fig. 1i).

### Intra-articular injection of S100A8 protein induces expression of monocyte markers in the synovium

**Fig. 2:**
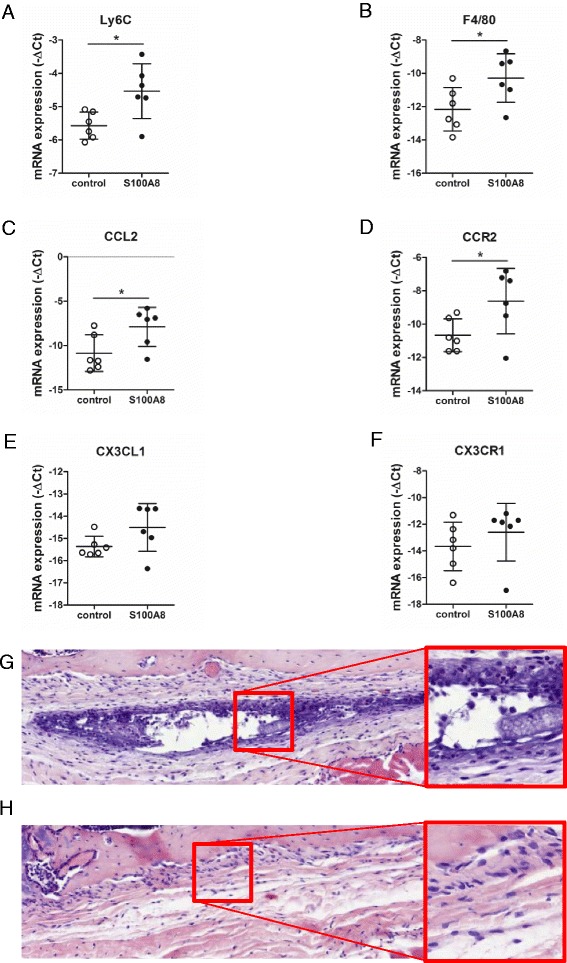
Intra articular injection of S100A8 leads to local induction of monocytes in the synovium. **a**-**f** mRNA expression of monocyte marker Ly6C (**a**), macrophage marker F4/80 (B), Ly6C^high^ chemokine CCL2 (**c**), and Ly6C^high^ chemokine receptor CCR2 (**d**) are increased in the synovium of mice 24 h after intra articular injection with S100A8, compared to BSA-injected controls, whereas Ly6C^low^ chemokine CX3CL1 (**e**), and Ly6C^low^ chemokine receptor CX3CR1 (**f**) are not affected after S100A8 administration, as analyzed using RT-qPCR. Data represent mean ± SD of six individual mice. *Significantly different from treated control (*p < 0.05). **g**, **h** HE staining on histological sections of the joint 24 h after intra-articular (i.a.) injection with S100A8 demonstrated strong presence of inflammatory cells with monocyte-like morphology in the synovial lining layer of the knee joints (**g**), whereas BSA injection as control resulted in less pronounced presence of inflammatory cells (**h**). Ct cycle threshold

Since influx of Ly6C^high^ monocytes coincides with high S100A8/A9 levels, we next investigated whether S100A8/A9 is able to regulate monocyte influx. We injected S100A8 (5 μg), which is described to be the most dominant form of the S100A8/A9 complex in mice [21], or BSA as control, into the knee joint of naive mice and determined the expression of several markers involved in monocyte attraction in the synovium after 24 h, using RT-qPCR (Fig. 2a-f). To get an impression whether monocytes are present, we first measured mRNA levels of Ly6C and F4/80. Significant upregulation of both Ly6C and F4/80 (2.1-fold and 3.7-fold, respectively) was observed (Fig. 2a and b).

mRNA levels of the Ly6C^high^ monocyte-attracting chemokine CCL2 and its receptor CCR2 were significantly elevated (7.8-fold and 4.1-fold, respectively) after S100A8 injection (Fig. 2c and d). In contrast, mRNA levels of the Ly6C^low^-attracting chemokine CX3CL1 and its receptor CX3CR1 were lower and were not statistically significant (1.8-fold and 2.1-fold, respectively) (Fig. 2e and f). To verify that monocytes are indeed recruited to the joint in response to S100A8 injection, we stained sections of the joints with HE 24 h after S100A8 injection. Figure 2g clearly shows the strong presence of inflammatory cells exhibiting typical monocyte morphology, which resided particularly in the synovial lining layer. This influx of inflammatory cells was absent in BSA-injected controls (Fig. 2h).

### Locally induced CiOA leads to a significant S100A8/A9-driven decrease of Ly6C^high^ monocytes in the BM, but not in the blood and spleen

As injection of S100A8 into the mouse knee joint leads to attraction of Ly6C^high^ monocytes particularly, we next investigated the role of S100A8/A9 on monocyte subpopulations during early CiOA in more detail. To this end, we used S100a9^-/-^ mice, which functionally are double knockout for S100A8 and S100A9, since S100A8 is missing at protein level as well [37].

**Fig. 3:**
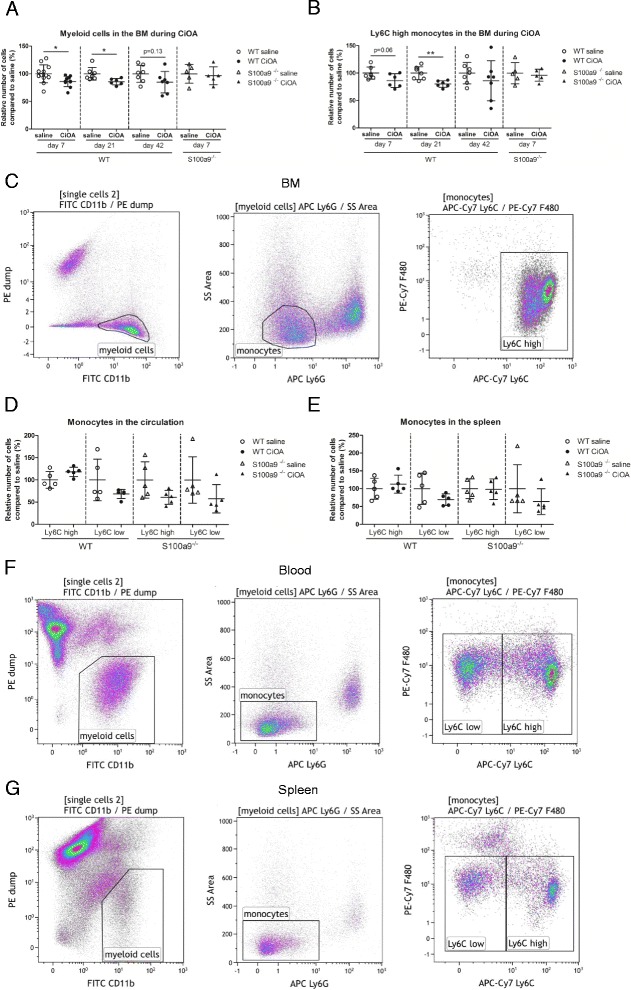
The number of Ly6C^high^ monocytes decreases during collagenase-induced osteoarthritis (CiOA) in the bone marrow (BM) of wild-type (WT) but not S100a9^-/-^ mice, but not in the blood and spleen of WT or S100a9^-/-^ mice. **a** The relative number of myeloid cells in the complete contralateral femur of WT mice was decreased early during CiOA compared to saline-injected controls, whereas no effects were found in S100a9^-/-^ mice at CiOA day 7. **b** The relative number of Ly6C^high^ monocytes in the complete contralateral BM of WT mice was also decreased early during CiOA compared to saline-injected control animals, whereas no effect was observed in S100A9^-/-^ mice. **c** Gating strategy of monocyte subsets in the BM, using flow cytometry. **d**, **e** The relative number of Ly6C^high^ and Ly6C^low^ monocyte subpopulations was not affected in the peripheral blood (**d**), and spleen (**e**) at CiOA day 7 in WT or S100a9^-/-^ mice when compared to saline-injected control animals. **f**, **g** Gating strategy of monocyte subsets in the peripheral blood (**f**) and spleen (**g**), measured using flow cytometry. Data represent mean ± SD of 5–7 individual mice per group (except for WT mice on day 7 where n = 11–12 animals). *Significantly different from saline-injected control (*p < 0.05, **p < 0.01, ***p < 0.001)

Locally induced inflammation in the joint may have an impact on migration of monocytes from the BM into the blood, and subsequently to the inflamed joint. Local induction of CiOA resulted in elevated levels of S100A8/A9 (up to 882 ng/mL and on average 502 ng/mL) in the blood at day 7 (Additional file 1: Figure S1) and induced systemic effects within the BM of WT mice. Measuring the total number of myeloid cells within a complete femur at several time points after induction of OA in WT mice demonstrated a reduction in myeloid cell number of 14% (*p* = 0.0210), 14% (*p* = 0.0205), and 15% (*p* = 0.1335) at day 7, 21, and 42 after CiOA induction, respectively (Fig. 3a), corresponding to release of Ly6C^high^ monocytes at day 7 (*p* = 0.0616), day 21 (*p* = 0.0036), and day 42 (*p* = 0.3982) (Fig. 3b), which only was significant at day 21, as determined by flow cytometry (Fig. 3c). In contrast, no changes in absolute myeloid cell numbers or Ly6C^high^ monocytes were observed in the femurs of S100a9^-/-^ mice at CiOA day 7 when compared to their saline-injected controls. This suggests that S100A8/A9 stimulates the efflux of monocytes from the BM into the blood. However the increase of monocytes into the blood at this time-point had no effect on the ratio of Ly6C^high^ and Ly6C^low^ subsets in both the blood (Fig. 3d) and in the spleen (Fig. 3e). No difference was found at day 7 CiOA when compared to saline injected controls, and also not between WT and S100a9^-/-^ mice (see Fig. 3f and g for the monocyte gating strategy of the blood and spleen, respectively, using flow cytometry).

### Increased numbers of Ly6C^low^, but not Ly6C^high^ monocytes in the synovium of day 7 CiOA in S100a9^-/-^ mice

**Fig. 4:**
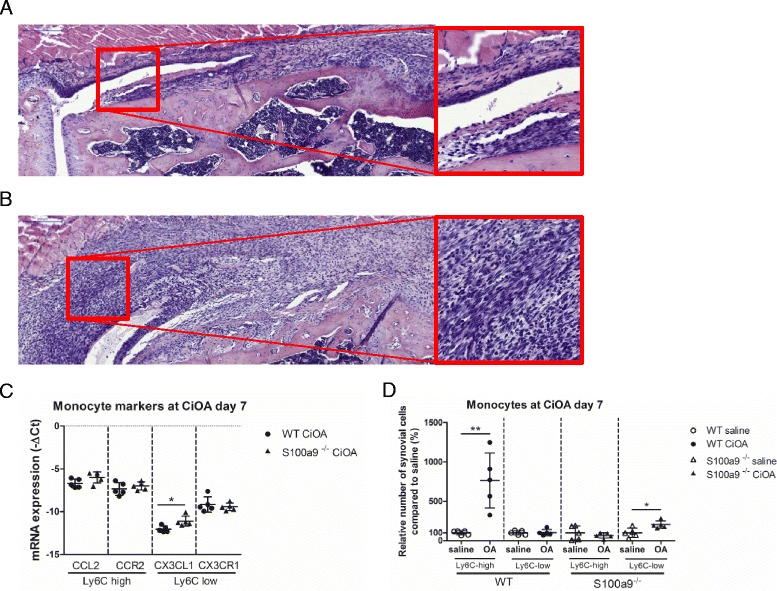
S100a9^-/-^ mice have locally more Ly6C^low^ monocytes in early collagenase-induced osteoarthritis (CiOA), compared to wild-type (WT) mice at CiOA day 7. **a**, **b** Less influx of inflammatory cells in S100a9^-/-^ (**a**) mice compared to WT mice (**b**) at CiOA day 7, as shown by HE staining. **c** mRNA expression of the Ly6C^low^ monocyte-subset-attracting chemokine CX3CL1 is increased in the synovium of S100a9^-/-^ mice compared to WT mice at CiOA day 7, while Ly6C^high^ monocyte-subset-attracting chemokine CCL2 and both monocyte subset cell markers CCR2 (Ly6C^high^) and CX3CR1 (Ly6C^low^) were not different between the two strains. **d** Relative number of Ly6C^high^ monocytes are increased locally in the joint of WT mice, whereas Ly6C^low^ monocytes are increased in S100a9^-/-^ mice, compared to saline-injected controls, as analyzed by flow cytometry. Data represent mean ± SD of five individual mice per group. *Significantly different from saline-injected control (*p < 0.05, ***p < 0.001)

As we observed differences in BM efflux between WT and S100a9^-/-^ mice, we additionally studied whether there also was a difference in cell influx into the synovium between these two strains. At day 7 after induction of CiOA in S100a9^-/-^, a lower number of inflammatory cells with monocyte morphology was observed in histological sections within the synovium when compared to WT mice (Fig. 4a vs WT b). We next studied mRNA expression of monocyte subset chemokine receptors and their ligands within the CiOA synovium of S100a9^-/-^ mice (Fig. 4c). Expression levels of Ly6C^high^ monocyte chemokine CCL2 and its receptor CCR2 were similar in S100a9^-/-^ and WT mice at CiOA day 7. In contrast, Ly6C^low^ chemokine CX3CL1 was significantly increased (1.9-fold) in S100a9^-/-^ mice at CiOA day 7, whereas the mRNA expression of Ly6C^low^ monocyte chemokine receptor CX3CR1 was similar in WT and S100A9 mice at CiOA day 7.

Finally, synovium obtained from S100a9^-/-^ mice at CiOA day 7 was shortly treated with collagenase and the numbers of Ly6C^high^ and Ly6C^low^ monocytes in the synovium were analyzed by flow cytometry (Fig. 4d). At day 7 CiOA in WT mice we found a significant rise in Ly6C^high^ monocytes, whereas Ly6C^low^ were not altered when compared to saline-injected WT mice. Interestingly, in S100a9^-/-^ mice, levels of Ly6C^high^ monocytes were not altered when compared to saline-injected controls, whereas there was a significant increase in Ly6C^low^ monocytes (205%).

Taken together, locally induced S100A8/A9 production in early OA causes influx of Ly6C^high^ monocytes particularly, which are responsible for early production of cytokines and tissue damage. Moreover, inhibition of S100A8/A9 might give preference to influx of Ly6C^low^ monocytes, thereby regulating repair.

## Discussion

Recent studies have shown that synovitis significantly contributes to the development of pathologic change in the joint during inflammatory osteoarthritis [6]. Previously, we demonstrated that the alarmin S100A8/A9 plays an important role in synovitis and joint pathologic change in CiOA [22]. In the present study, we further explored the involvement of S100A8/A9 in the recruitment of monocyte subpopulations towards the synovium in CiOA. Our findings suggest that prolonged S100A8/A9 production during induction of inflammatory OA locally leads to strongly elevated mobilization of predominantly Ly6C^high^ monocytes into the joint in OA and from the BM.

The monocyte is the major inflammatory cell type within the synovium throughout the course of inflammatory OA [13–15]. In the mouse, two types of monocytes have been described. Ly6C^high^ monocytes, expressing high levels of CCR2 and guided by chemokine CCL2 are involved in removing tissue debris and produce mainly pro-inflammatory cytokines like IL-1β and S100A8/A9. In contrast, Ly6C^low^ monocytes, expressing high levels of CX3CR1 and attracted by CX3CL1, release anabolic factors like VEGF and TGF-β, involved in repairing joint tissue [31, 32].

The relative number and the interplay between Ly6C^high^ and Ly6C^low^ monocytes within the synovium determine the severity of inflammation and regulate further development of pathologic change in the tissues. Previous studies in our laboratory showed that selective removal of resident lining macrophages prior to induction of CiOA largely inhibited monocyte cell influx and development of joint destruction [40, 42].

In OA, resident synovial macrophages that cover the surface of the synovium are the first cells cleaning the extracellular matrix (ECM) fragments released by damaged joint tissue [43]. These fragments are recognized by TLRs and scavenger receptors, leading to activation of synovial macrophages thereby releasing high levels of CCL2. This elevates influx of Ly6C^high^ monocytes favoring further removal of tissue debris.

On the other hand, a shift towards higher numbers of Ly6C^high^ monocytes within the inflamed synovium may foster pathologic change in the joint by elevated release of pro-inflammatory cytokines. However, only low levels of cytokines like TNF-α, IL-1β, and IL-6 were observed during the first phase of CiOA and probably are only marginally involved in driving pathologic change in the joint. Induction of CiOA in mice lacking IL-1α/β had no effect on either synovitis or on joint pathology [44]. The outcome was similar using IL-6^-/-^ and TNF-α^-/-^ mice (manuscript in preparation). In contrast, alarmin S100A8/A9 was very strongly upregulated within the synovium and measured in significant amounts for prolonged periods up till day 42 after CiOA induction. The heterodimer is released by monocytes and activated macrophages and stimulates nearby synovial cells via TLR4 to release chemokines that attract monocyte populations [45, 46].

In the present study we found that levels of CCL2, attracting Ly6C^high^ monocytes, are much higher in day 7 CiOA compared to saline-injected controls in WT mice, resulting in increased presence of Ly6C^high^ monocytes in the synovium. In addition, CCL2 expression was strongly enhanced in the synovium after i.a. injection of S100A8, pointing towards an important role for S100A8/A9 in this process. Whereas in synovium at day 7 CiOA, expression of CCR2/CCL2, characteristic of Ly6C^high^ monocytes, was comparable in both WT and S100a9^-/-^ mice, the Ly6C^low^-attracting chemokine CX3CL1 and Ly6C^low^ monocyte population was raised in the synovium of S100a9^-/-^ mice. Since Ly6C^high^ monocytes were not elevated in S100a9^-/-^ synovium, but the Ly6C^low^ monocytes were, S100A8/S100A9 may favor the presence of Ly6C^high^ monocytes within the inflamed synovium, not only by chemotactic attraction but also by suppressing their differentiation into the Ly6C^low^ population thereby maintaining the monocyte population into a more pro-inflammatory state.

Recently it was shown that monocytes have a memory, which is triggered by TLR4 ligands like lipopolysaccharide (LPS) [47]. This memory is initiated by epigenetic programming and may drive monocytes into a cell type that sustains its pro-inflammatory characteristics [48]. S100A8/A9 is an important TLR4 ligand released in high levels for prolonged periods in the joint in OA and may be a major mediator driving this epigenetic programming. Ly6C^high^ monocytes are high producers of S100A8/A9, which may form a positive feedback loop. Finally Ly6C^high^ and Ly6C^low^ monocytes are possibly able to differentiate into M1-like and M2-like macrophages, respectively [49, 50]. The local environment within the synovium strongly influences the signature of the monocytes and their differentiation into their mature forms [50, 51].

BM and spleen form important reservoirs for Ly6C^high^ monocytes, which become available under inflammatory conditions. Local induction of CiOA causes high levels of S100A8/A9 within the synovium for prolonged periods; up to 5 μg/mL have been measured in synovial washouts of CiOA synovium [52]. These proteins leak from the joint into the blood and elevated levels of S100A8/A9 levels were measured throughout the course of OA until the endpoint at day 42. Other pro-inflammatory cytokines like IL-1β, TNF-α, or IL-6 were not detected within the blood.

Interestingly local induction of CiOA in the knee joints of WT mice induced a significant efflux of Ly6C^high^ monocytes from the BM into the blood when compared to saline-injected WT mice, which was absent at CiOA day 7 in S100a9^-/-^ mice. This indicates that low-grade local joint inflammation in OA is able to initiate release of Ly6C^high^ monocytes from the BM. This is in agreement with an earlier study showing that locally induced low-grade inflammation in the heart in myocardial infarction [32, 53] induced efflux from the BM resulting in elevated local influx of Ly6C^high^ monocytes within the lesions. Which mechanisms that are initiated within the inflamed joint drive monocyte efflux from the BM is momentarily under investigation. In contrast to conventional pro-inflammatory cytokines, which were undetectable in the blood, S100A8/A9 levels were very high during the course of CiOA. S100A8/A9 in the blood is not only transported as a free protein but also inside extracellular vesicles (EVs). EVs, released by activated immune cells like monocytes/neutrophils, have been shown to contain S100A8/A9 [54, 55]. They are released at local inflammatory sites and additionally transported to distant areas where they can affect other cells by either fusion with their membrane or when recognized by receptors [54, 55]. In addition, mechanisms like sympathetic nerve signaling may explain part of the systemic effects observed in the BM. Earlier studies suggested that myocardial infarction liberates monocytes from the BM due to sympathetic nerve signaling thereby boosting atherosclerosis in which pathologic change is highly regulated by the influx of monocyte subpopulations [53].

Myeloid precursors in the BM differentiate into Ly6C^high^ monocytes driven by growth factors like PU.1, and M-CSF. Local inflammation promotes the release of Ly6C^high^ monocytes from the BM into the circulation where they either remain in that state or are transformed into Ly6C^low^ monocytes [32]. Interestingly, S100a9^-/-^ mice did not have elevated efflux of monocytes at CiOA day 7. S100A8/A9 is also abundantly expressed within the BM. Systemic inflammatory triggers and/or sympathetic nerve signaling may promote local release of S100A8/A9 heterodimer within the BM stimulating migration of Ly6C^high^ monocytes to the blood. This is supported by our findings that expression levels of LFA-1 (Integrin-β2), VCAM, L-selectin, PECAM1, and VE-cadherin were significantly decreased in the BM of S100a9^-/-^ mice (Additional file 1: Figure S2). It is described that all these adhesion molecules contribute to leucocyte adhesion and migration in the BM [34, 56]. S100A8/A9 alarmins are strong activators of the beta-2 integrin on myeloid cells and have been shown to be important in transendothelial migration of phagocytes [26, 57–59]. Moreover, previous studies have already shown that S100A8/A9 drives primary BM expansion of myeloid-derived suppressor cells (MDSC) driven by the S100A9/CD33 pathway thereby altering hematopoiesis [60].

Apart from the BM the spleen also forms an important reservoir for storage of Ly6C^high^ monocytes [61] and may contribute to sustaining synovitis in OA. Although it is described that acute inflammation promotes monocyte release from the spleen [62], we did not find any effect on the ratio of monocyte populations in the spleen of mice at CiOA day 7. This is in agreement with an earlier study in which it was found that in a more severe model of arthritis, splenectomized mice did not differ in ankle swelling or clinical score compared to non-splenectomized mice [63], suggesting that monocytes released from the spleen do not contribute to synovial inflammation in arthritis.

Although BM efflux of monocytes was significantly raised at CiOA day 7, no effect on the ratio of monocyte subsets was observed within the blood. Although the data as presented in this manuscript strongly suggest a role for S100A8/A9 in the efflux of cells from the BM and into the inflamed joint, we cannot prove a causal relation for this mechanism. Monocytes are released from the BM as Ly6C^high^ monocytes and the ratio of Ly6C^high^ and Ly6C^low^ may be rapidly balanced by systemic factors. Tracking studies using fluorescent-labeled monocytes are in progress and will answer to what extent Ly6C^high^ monocytes released from the BM are able to reach the joint in OA.

OA is characterized by cartilage and bone destruction, which is related to synovitis [6]. A privileged presence of Ly6C^high^ monocytes within the synovium leads to prolonged production of inflammatory cytokines (particularly S100A8/A9) and proteases, which may contribute to joint destruction. Earlier studies showed that S100A8/A9 is crucial in mediating cartilage and bone destruction within the CiOA model [22]. S100A8/A9 stimulates chondrocytes to produce matrix metalloproteinases (MMPs) thereby degrading the surrounding ECM [64], which drives synovitis in a positive feedback loop. In addition, S100A8/A9 directly stimulated osteoclastogenesis and strongly increased osteoclast-mediated bone destruction. The human analogs of Ly6C^high^ and Ly6C^low^ in the mouse are classical CD14++CD16- and non-classical CD14 + CD16+, respectively. Investigating the balance of these monocyte subsets within inflammatory OA in synovium and BM may give more insight into how synovitis and joint destruction are connected to this crippling disease.

In summary we found that S100A8/A9 is a crucial alarmin involved in favoring recruitment of Ly6C^high^ monocytes into the CiOA knee joint. Local production of S100A8/A9 attracts Ly6C^high^ monocytes and may suppress their differentiation into Ly6C^low^ monocytes. Moreover production of S100A8/A9 within the bone marrow may further promote efflux of Ly6C^high^ monocytes into the circulation. Ly6C^high^ monocytes are potent producers of S100A8/A9 keeping the monocyte population in a pro-inflammatory state and forming a positive feedback loop. Understanding the underlying inflammatory process in the synovium in OA may lead to new therapeutic targets and inhibiting S100A8/A9 may be an interesting therapeutic target to improve the outcome of OA pathology.

## Conclusions

Induction of OA leads to the elevation of S100A8/A9 locally in the joint and systemically in the circulation, and leads to the mobilization of Ly6C^high^ monocytes from the BM to the joint. S100A8/A9 has an important role in driving OA pathology, probably by regulating the local environment in the joint determining whether Ly6C^high^ or Ly6C^low^ monocytes are recruited.

## Additional file

Additional file 1: Figure S1. S100A8/A9 protein levels are systemically elevated in the serum during early CiOA. Systemic levels of S100A8/A9 protein in serum of WT mice are increased early during CiOA compared to saline-injected control mice, measured using ELISA. Data represent mean ± SD of five individual mice per group per time point. *Significantly different from saline-injected control (**p* < 0.05, ***p* < 0.01, ****p* < 0.001). **Figure S2.** Expression of adhesion molecules is lower in the BM of WT mice, compared to S100a9^-/-^ mice, at CiOA day 7. mRNA expression of several adhesion molecules is lower in the BM of S100a9^-/-^ mice compared to WT mice at CiOA day 7. Data represent mean ± SD of five individual mice. *Significantly different from saline-injected control (**p* < 0.05, ***p* < 0.01, ****p* < 0.001). (PPT 142 kb)

## Abbreviations

bp: Base pair (s)
BM: Bone marrow
BSA: Bovine serum albumin
cDNA: Complementary DNA
CCL2: (C-C motif) ligand
CCR2: C-C chemokine receptor type 2
CiOA: Collagenase-induced osteoarthritis
Ct: Cycle threshold
DAMPs: Damage-associated molecular patterns proteins
ELISA: Enzyme-linked immunosorbent assay
EVs: extracellular vesicles
FACS: Fluorescence-activated cell sorting
HE: Haematoxylin and eosin
IL: Interleukin
MRP: Myeloid related proteins
NK: Natural killer
OA: Osteoarthritis
PBS: Phosphate-buffered saline
RT-qPCR: Reverse-transcription quantitative PCR
TGF: Transforming growth factor
TLR: Toll-like receptor
TNF: Tumor necrosis factor
VEGF: Vascular endothelial growth factor
WT: Wild-type
