# Supplementary figures and images for: S100A8/A9 increases the mobilization of pro-inflammatory Ly6Chigh monocytes to the synovium during experimental osteoarthritis
Source: Arthritis Res Ther. 2017 Sep 29;19:217. doi: 10.1186/s13075-017-1426-6 (PMC5623958; doi:10.1186/s13075-017-1426-6)

## Slide 1
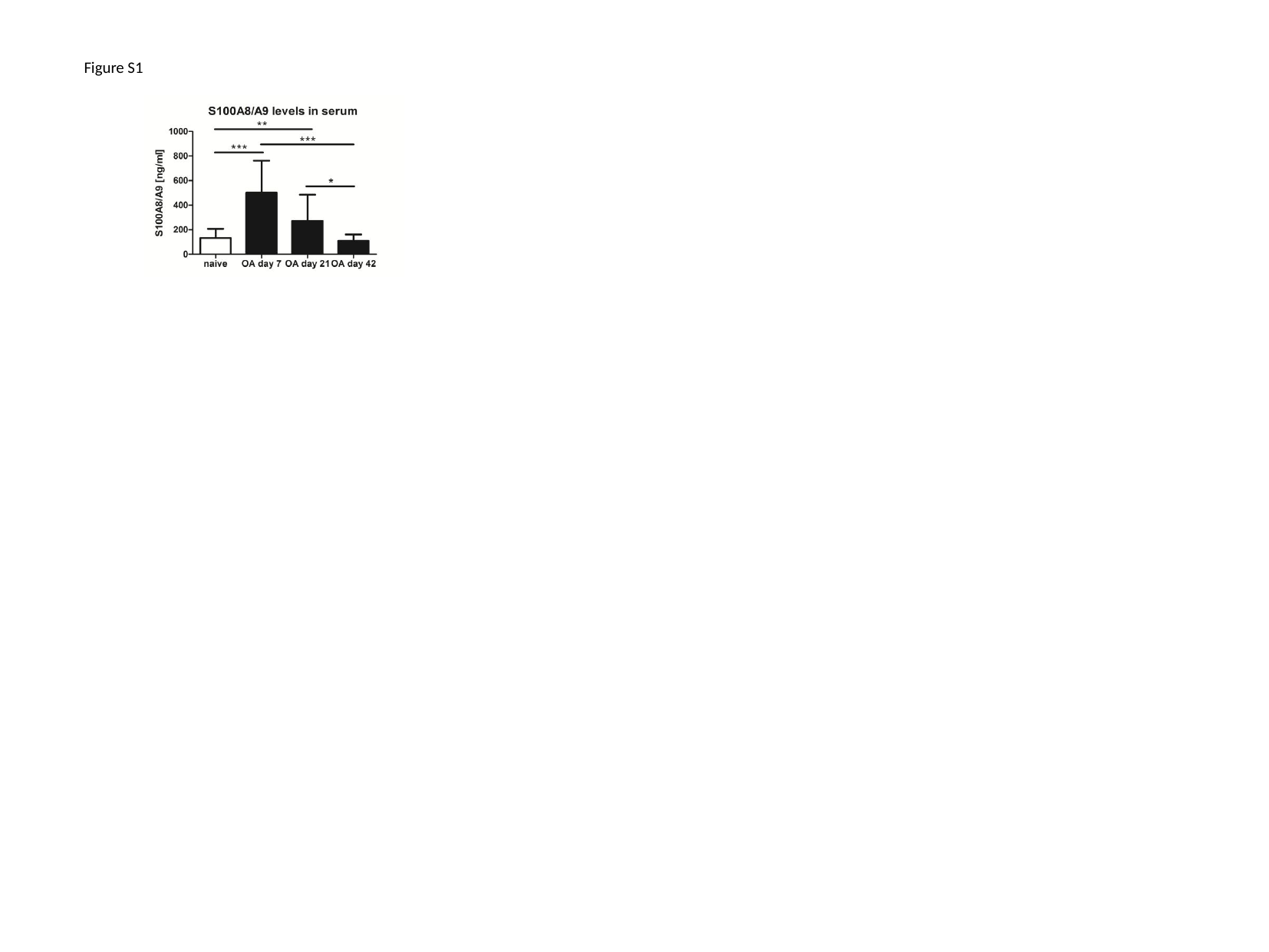

Figure S1

## Slide 2
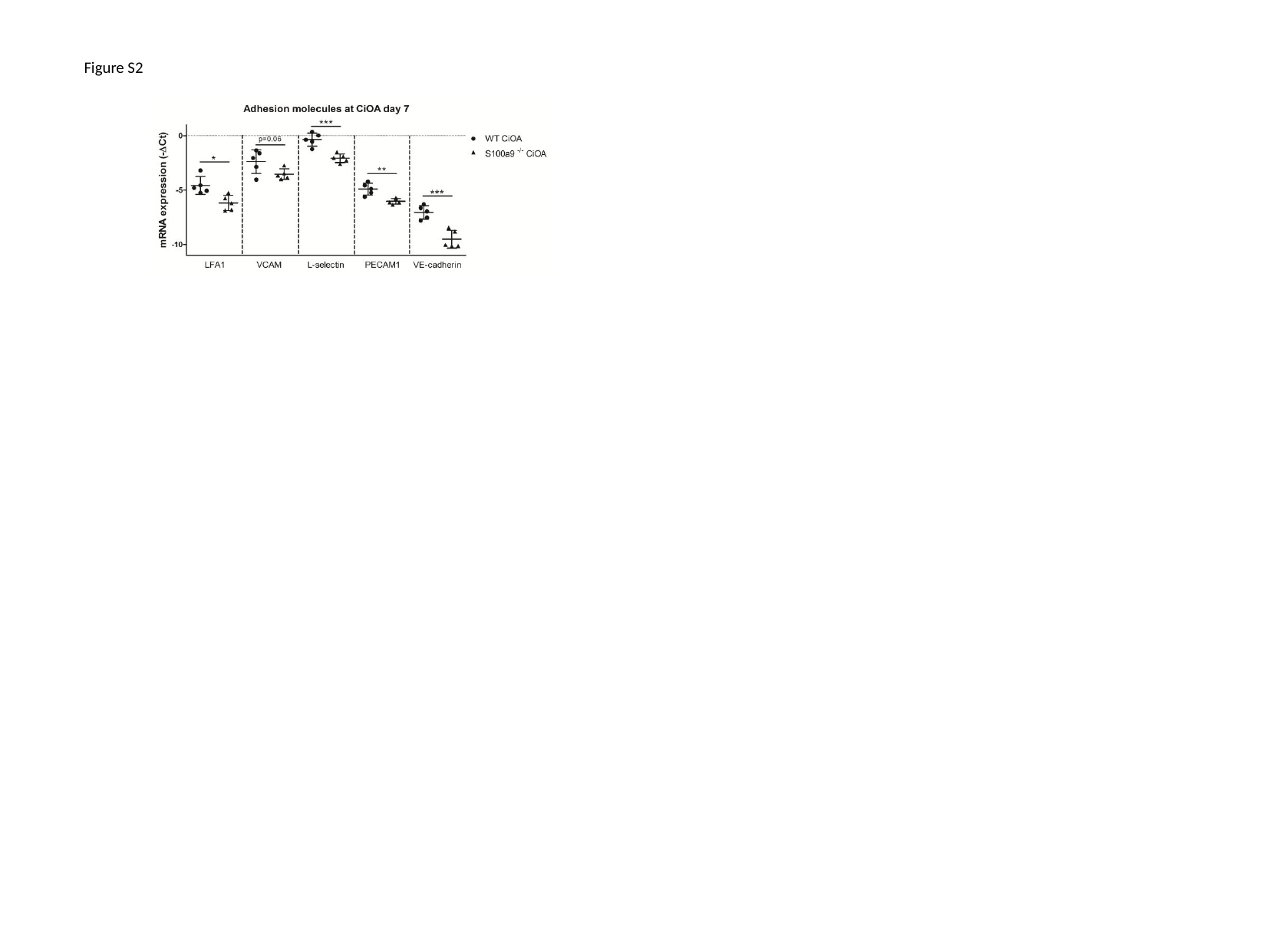

Figure S2

Supplement: Additional file 1: Figure S1. — S100A8/A9 protein levels are systemically elevated in the serum during early CiOA. Systemic levels of S100A8/A9 protein in serum of WT mice are increased early during CiOA compared to saline-injected control mice, measured using ELISA. Data represent mean ± SD of five individual mice per group per time point. *Significantly different from saline-injected control (*p < 0.05, **p < 0.01, ***p < 0.001). Figure S2. Expression of adhesion molecules is lower in the BM of WT mice, compared to S100a9-/- mice, at CiOA day 7. mRNA expression of several adhesion molecules is lower in the BM of S100a9-/- mice compared to WT mice at CiOA day 7. Data represent mean ± SD of five individual mice. *Significantly different from saline-injected control (*p < 0.05, **p < 0.01, ***p < 0.001). (PPT 142 kb) [file 13075_2017_1426_MOESM1_ESM.ppt]
